# Effect of PEG-600 Incorporation on the Mechanical and Thermal Response of Tunable Fiber-Reinforced Shape Memory Polymer Composites

**DOI:** 10.3390/polym17202742

**Published:** 2025-10-14

**Authors:** Marylen T. De la Cruz, Riana Gabrielle P. Gamboa, Ricky Kristan M. Raguindin, Jon Dewitt E. Dalisay, Eduardo R. Magdaluyo

**Affiliations:** 1 Department of Mechanical Engineering, College of Engineering, University of the Philippines Diliman, Quezon City 1101, Philippines; 2 Department of Mining, Metallurgical and Materials Engineering, College of Engineering, University of the Philippines Diliman, Quezon City 1101, Philippines

**Keywords:** shape memory polymer composites, epoxy resin, carbon–aramid fibers, PEG-600, thermomechanical properties, glass transition temperature

## Abstract

Shape memory polymer composites (SMPCs) are an intelligent class of materials capable of self-actuation, offering promising applications in diverse stimuli-responsive material systems. This study developed epoxy-based SMPCs reinforced with carbon–aramid fibers at a 15:85 ratio, with their glass transition temperature (T_g_) tailored by incorporating 5 wt.% (SMPC-5) and 10 wt.% (SMPC-10) polyethylene glycol (PEG-600). Dynamic mechanical analysis (DMA) confirmed that PEG addition effectively reduced the T_g_ from 89.79 °C in the neat composite (SMPC-P) to 70.28 °C in SMPC-5 and 59.34 °C in SMPC-10. Incorporating 5 wt.% PEG enhanced storage and loss moduli, whereas excessive plasticization at 10 wt.% reduced stiffness. Infrared spectroscopy analysis revealed shifts and increased intensities in hydroxyl (OH), aliphatic C-H, and carbonyl (C=O) groups, indicating enhanced intermolecular interactions and bond formation. Tensile testing showed that the carbon–aramid filler significantly improved tensile strength and stiffness, with SMPC-10 achieving the highest tensile strength (233.59 MPa) and SMPC-5 the highest Young’s modulus (14.081 GPa). These results highlight the complementary role of carbon–aramid reinforcement and PEG plasticization in tuning thermomechanical behavior, providing baseline insights for designing SMPCs with tailored actuation and reliable structural performance.

## 1. Introduction

Shape memory polymers (SMPs) are an emerging class of smart materials with the unique ability to self-actuate. When triggered by external stimuli such as heat or light, they can be deformed into a temporary shape and subsequently recover their original configuration under specific conditions [1]. Compared with traditional materials, SMPs exhibit several advantages, including their low density, affordability, large deformability, adjustable transition temperature, and ease of production [2].

Throughout the years, shape memory polymers (SMPs) have garnered significant attention as potential alternatives to shape memory alloys (SMAs) due to their cost-effectiveness and significantly lower density [3]. However, their practical applications are constrained by poor mechanical properties, limited recovery stress, low storage modulus, and low shape-recovery speed, underscoring the need for enhancement through the development of composite systems and modified matrices with improved performance across various applications.

**Figure 1:**
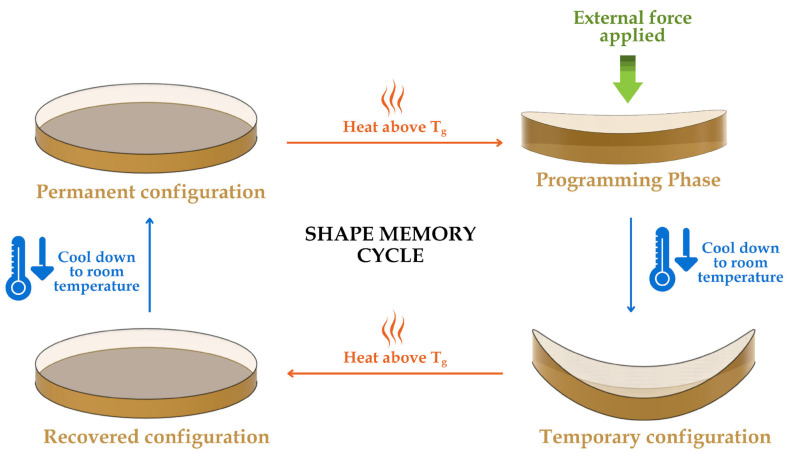
Shape programming sequence of a thermally induced epoxy-based SMP.

To address these challenges, SMP matrices are often reinforced with fillers to create shape memory polymer composites (SMPCs). This strategy aims to improve mechanical performance while maintaining shape-memory functionality. A primary approach involves reinforcing fillers that can take the form of either particles or fibers [4]. Of these two, continuous fiber reinforcements have demonstrated superior performance in high-strength applications by significantly improving strength and stiffness, as well as providing excellent resistance against creep and relaxation. Their intrinsic properties also play a crucial role in determining the most effective activation method for the composite system. Commonly used SMP matrices in SMPCs include epoxy, cyanate ester, polyurethane, polyimide, polystyrene, bismaleimide, and polyester [5]. Epoxy is particularly valued for its superior mechanical properties and precise control of shape-memory behavior [6]. Thermally induced epoxy-based SMPs undergo three critical steps that produce a one-way shape memory effect: (1) heating above T_g_ to reshape the material into a temporary form, (2) cooling under an external force to lock polymer chains in that configuration, and (3) reheating to release stored stress, and energy that allows the polymer material to its original shape as shown in Figure 1 [7].

As an early attempt to explore this field, Liang et al. [8] reported that woven fiberglass SMP composites achieved a 50% increase in ultimate strength and a fourfold improvement in modulus compared with neat SMPs. These findings established a foundation for subsequent investigations into the potential of different reinforcement materials and novel fabrication techniques. More recently, Li et al. [9] evaluated the performance of unidirectional carbon fiber-reinforced SMPs with fiber mass fractions of 16%, 23%, 30%, and 37% across a series of three-point bending tests. Their findings provide valuable insights into how reinforcement type, mass fraction, and laminate sequence can guide the design of future SMPC systems. In addition, SMP performance can also be tailored through matrix modification using plasticizers or additives. For example, incorporating polyethylene glycol (PEG) has been shown to enhance thermomechanical properties by adjusting the glass transition temperature to suit specific applications and to help the balance between flexibility with strength [10].

In line with current approaches to enhance the performance of shape memory polymers (SMPs), the present study focused on the development of an epoxy-based shape memory polymer composite reinforced with a distinct 15:85 ratio of epoxy resin to carbon–aramid fiber. To further tailor its functional properties, particularly the glass transition temperature (T_g_), the incorporation of polyethylene glycol (PEG-600) was varied at concentrations of 5 wt.% and 10 wt.%. This modification not only improved the flexibility and chain mobility of the polymer matrix but also enabled control over the thermal activation window of the composite, which is critical for targeted actuation and stability in practical applications.

While SMP composites have been extensively studied, limited research has explored epoxy–carbon–aramid systems with controlled glass transition characteristics. Addressing this gap, the synthesized composites in this study were characterized to establish a direct relationship between material composition, thermomechanical properties, and overall performance. Fourier-transform infrared spectroscopy (FTIR) was employed to verify chemical interactions and confirm the integration of PEG within the epoxy network. Dynamic mechanical analysis (DMA) provided quantitative insights into viscoelastic behavior and T_g_ shifts as a function of PEG loading, while tensile and flexural properties were evaluated using a universal testing machine (UTM). To complement the experimental data, finite element simulations in ANSYS were conducted to visualize stress distribution and deformation mechanisms under loading, thereby bridging experimental observations with predictive modeling.

The findings of this work not only provide baseline insights into the design of SMP composites with controlled actuation behavior but also highlight the feasibility of tailoring epoxy–carbon–aramid systems for specialized functions. The results indicate the importance of synergistic reinforcement and matrix modification in achieving targeted thermomechanical responses. This approach is crucial for applications requiring precise thermal activation, high strength-to-weight ratios, and durability under cyclic loading. Ultimately, this study supports the design of smart polymer composite systems for potential use in aerospace and adaptive structural components.

## 2. Materials and Methods

### 2.1. Material Specifications

**Figure 2:**
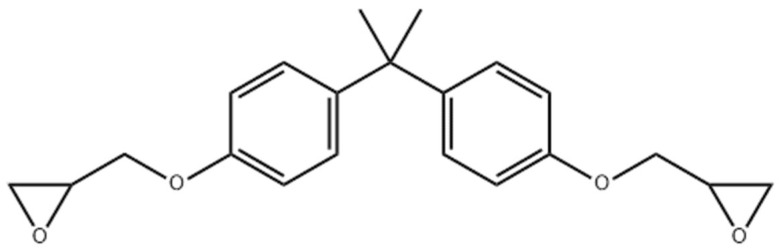
The chemical structure of DGEBA.

The SMPC specimens were fabricated from a shape memory polymer (SMP) matrix combined with carbon–aramid hybrid fiber fillers. The polymer matrix employed Polytech Laminating Epoxy (Polymer Products Philippines, Inc., Manila, Philippines), a two-component, non-solvent system. Component A is the epoxy resin, Bisphenol A diglycidyl ether (DGEBA), a liquid product synthesized through the reaction of epichlorohydrin with bisphenol A, and having the molecular formula C_21_H_24_O_4_. Figure 2 presents the molecular structure diagram of this compound. Component B, on the other hand, is a curing agent or hardener formulated with modified amidoamine. Table 1 provides a comprehensive list of the different material properties relevant to the tests conducted.

The reinforcement filler consisted of a hybrid woven fabric procured from Polymer Products Philippines, Inc. It featured a 2 × 2 twill weave configuration, with aramid fibers serving as the primary reinforcement and carbon fibers as the secondary. This hybrid composition creates a complementary reinforcement system for this application by combining the high impact and fracture resistance of aramid fibers with the high tensile strength and stiffness of carbon fibers. The pertinent physical properties of the reinforcement filler are summarized in Table 2.

### 2.2. Fabrication of SMPCs

#### 2.2.1. Formation of Sheet-Metal Molds

Rectangular molds with dimensions of 15 mm × 30 mm were fabricated from sheet metal for sample preparation. Flat sheet metal pieces were also cut from the same stock and positioned on top of the shape memory polymer and carbon fiber hand-layup assembly during the curing process. This method introduced uniform pressure on the surface, which eliminated entrapped bubbles and helped maintain consistency of the sample thickness. In addition, the edges and corners were sealed with epoxy steel to prevent any resin spillage.

#### 2.2.2. Preparation of SMP Matrix

A total of three formulations were developed: (i) neat SMP, (ii) SMP containing 5% PEG-600, and (iii) SMP containing 10% PEG-600. Polyethylene glycol (PEG-600) was used as a plasticizer to increase the free volume between polymer chains. This additive reduces the stiffness of the polymer matrix and facilitates an earlier transition from the glassy to the rubbery state [10].

The selection of polyethylene glycol (PEG-600) was a critical design choice due to its unique balance of properties. Unlike lower molecular weight PEGs, which can volatilize during the curing process or cause phase separation, PEG-600 remains homogeneously mixed with the epoxy network, ensuring both miscibility and stability. Furthermore, its intermediate molecular weight provides better plasticization. In contrast to very-high-molecular-weight PEGs (e.g., PEG-1000) that can act as soft segments and drastically reduce the material’s modulus, PEG-600 efficiently lowers the glass transition temperature (T_g_) without compromising the structural integrity of the final product.

Components A and B of the epoxy resin were measured at a 2:1 (A: B) volumetric ratio and poured into separate plastic cups. Both were placed in a water bath maintained at 40 °C on a hot plate to reduce the viscosity of the epoxy components and to facilitate more effective mixing. After heating, the components were mixed vigorously by hand for one minute and then in a vortex shaker at 200 rpm for an additional minute. In the PEG-modified formulations containing 5% and 10%, the PEG-600 was first blended with Component A prior to combining it with component B. The final mixture was degassed in an ultrasonicator at room temperature until no visible bubbles remained.

#### 2.2.3. Addition of Carbon–Aramid Fiber Reinforcement

A constant carbon fiber-to-resin mass ratio of 15:85 was maintained across all samples. The fiber-to-resin ratio was restricted to 15 wt.% to isolate the effects of PEG-600 on shape memory behavior, and higher loadings and scalability tests are identified as areas for future work.

To create the SMPC, a layer of carbon–aramid fiber fabric was incorporated into the epoxy-based SMP matrix via the hand-layup method. Molds and flat metal sheets were coated with mold-release wax to ensure that the SMPC would not adhere to the mold walls upon curing. The SMP matrix was then poured into the mold and spread evenly across the bottom using a 3D-printed scraper. A 15 mm × 30 mm carbon–aramid fiber layer was cut and then placed on top of the polymer matrix. This layer was pressed in multiple directions to ensure complete fiber impregnation and to eliminate any trapped air pockets. The remaining SMP matrix was subsequently poured over the carbon fiber layer and spread evenly using the scraper. Once done, the flat metal sheet was lightly pressed on top to guarantee even distribution and consistent sample thickness. This process was applied for all three formulations to create the following SMPC samples: neat SMP with fiber reinforcement (SMPC-P), SMP containing 5% PEG-600 with fiber reinforcement (SMPC-5), and SMP containing 10% PEG-600 with fiber reinforcement (SMPC-10).

#### 2.2.4. SMPC Curing and Programming

The three molds containing the composites were placed in a Yamato DKN402 constant-temperature oven (Yamato Scientific Co., Ltd., Tokyo, Japan) for thermal curing. The samples were subjected to a three-step curing process consisting of initial curing at 80 °C for 2 h, secondary curing at 100 °C for 2 h, and final stabilization at 120 °C for 1 h. This multi-step curing sequence was adapted from the methodology presented by Li et al. [11] and further refined to align with the specific thermal characteristics of the selected epoxy resin system. After curing, the SMPC samples were carefully removed from the sheet metal molds, outlined and then cut to the specified dimensions using industrial-grade shears according to the requirements of subsequent tests to be conducted.

### 2.3. Material Characterization

#### 2.3.1. Dynamic Mechanical Analysis (DMA)

Dynamic mechanical properties of the three SMPC formulations were measured using the TA Instruments Q800 Dynamic Mechanical Analyzer (TA Instruments, New Castle, DE, USA) to obtain the loss modulus (E′), storage modulus (E″), and loss tangent (tan δ) versus temperature curves.

The samples were first prepared and cut to dimensions of 60 mm × 10 mm × 1 mm in accordance with the dimensional constraints of the testing equipment. A dual-cantilever clamp (ASTM D4065) was employed given its suitability for testing stiff thermoset polymers and composites [12]. This fixture also minimizes sample slippage and reduces measurement variability caused by unintended bending or shifting during testing.

An initial strain sweep was performed over a range of 0.1 to 10,000 μm to determine the linear viscoelastic region (LVR) of the material. The resulting data was used to identify the appropriate strain value for the following tests to ensure that the samples remained within their linear mechanical response range. Subsequently, a temperature ramp method was employed to determine the glass transition temperatures of the SMPC variants. The test was conducted over a temperature range of 30 °C to 150 °C, with a heating rate of 3 °C per minute. Throughout the test, a constant frequency of 1 Hz and a strain amplitude of 0.01% were maintained. From this test, key viscoelastic properties including storage modulus, loss modulus, and tan delta were obtained and analyzed using the TA Universal Analysis software (Universal Analysis v4.5A).

#### 2.3.2. Fourier Transform Infrared Spectroscopy (FTIR)

FTIR analysis was conducted in attenuated total reflectance (ATR) mode using a Thermo Scientific^TM^ Nicolet^TM^ iS50 FTIR Spectrometer (Thermo Scientific, Waltham, MA, USA). Neat SMP and all three SMPC formulations were tested to assess their molecular composition and to identify the functional groups present in the polymer matrix. This analysis also aimed to examine chemical bond formations related to the epoxy resin and fiber integration, and to evaluate the influence of PEG-600 incorporation and concentration. FTIR analysis was limited to qualitative comparisons of key absorption bands, with no quantitative peak-area integrations performed.

### 2.4. Mechanical Characterization

#### 2.4.1. Uniaxial Tensile Testing

Mechanical characterization of the fabricated samples was conducted using the uniaxial tensile testing machine Instron 3366 for the comparison of the behavior of neat SMP and SMPC samples under tensile loading. The test measured mechanical properties including ultimate tensile strength (UTS), Young’s modulus, stress–strain response and elongation at break.

**Figure 3:**
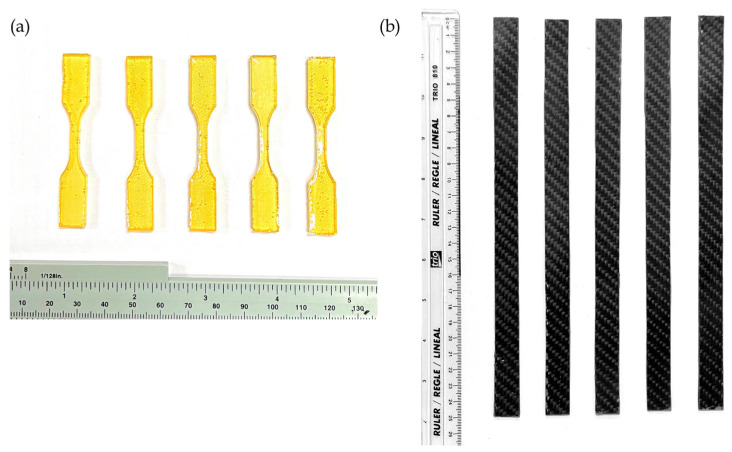
Fabricated SMP specimens following the ASTM D638 Type V standard (**a**) and SMPC samples following the ASTM D3039 standard (**b**).

The SMP test specimens, as shown in Figure 3a, were prepared in a dog-bone configuration, in accordance with the ASTM D638 Type V specifications, or the standardized test method for determining tensile properties of plastics [13]. The SMPC samples were fabricated following the dimensions provided by ASTM D3039 or the standardized test method for tensile properties of polymer matrix composite materials. Each SMPC sample was measured at 250 mm in length and 15 mm in width, as shown in Figure 3b. A total of 22 samples were tested, consisting of 7 neat SMPs and 15 carbon-reinforced SMPCs with varying PEG-600 contents. The aim was to assess the effect of hybrid fiber reinforcement on SMPs and the influence of PEG-600 concentration on the mechanical properties of SMPCs.

#### 2.4.2. ANSYS Simulations

A finite element analysis (FEA) model was developed in ANSYS 2023 R1, following the methodology of Jia et al. [14], to theoretically simulate the behavior of the SMPC samples. Before conducting the ANSYS simulation, the mechanical properties required for the model were determined through direct computation. The values derived from mechanical characterization tests were used in the calculations described below. This section followed the theory of macroscopic orthotropic elastic parameters to obtain four key parameters: (i) macroscopic elastic modulus of a single layer of composite material (E_1_/E_f_), (ii) macroscopic elastic modulus perpendicular to the fiber (E_2_), (iii) Poisson’s Ratio (v_12_), and (iv) shear modulus (G_12_). The corresponding formulas for determining these parameters are presented in Equations (1)–(5).

*Macroscopic elastic modulus of a single layer of composite material*

(1) $$E_{f}\;=\;c_{m}v_{m}\;+\;c_{f}v_{f}$$

*Macroscopic elastic modulus perpendicular to fiber*

(2) $$G_{12}=\frac{1}{c_{m}+c_{f}(E_{m}/E_{f})}$$

*Poisson’s Ratio*

(3) $$v_{21}\;=\;c_{m}v_{m}\;+\;c_{f}v_{f}$$

(4) $$v_{12}=\frac{E_{2}}{E_{1}}\;v_{12}$$

*Shear modulus*

(5) $$G_{12}=\frac{G_{m}G_{f}}{G_{f}c_{m}+G_{m}c_{f}}$$

where *c* is the volume fraction and *E* is Young’s modulus, with subscripts *m* denoting the properties of the SMP matrix and *f* denoting the properties of the carbon fiber.

**Figure 4:**
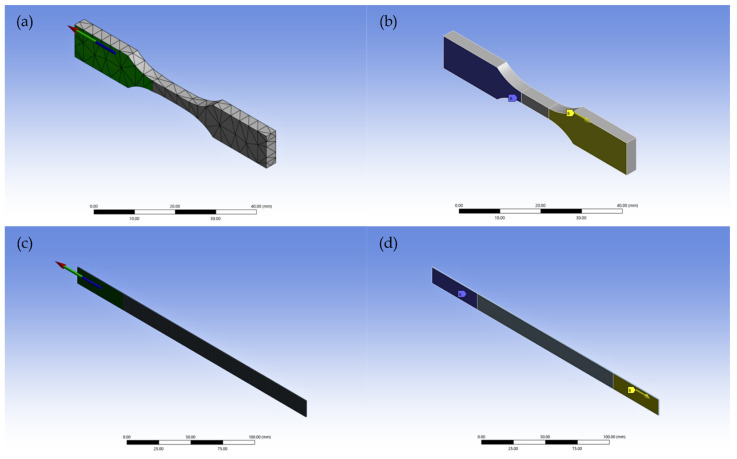
Finite element model setup of the SMP and SMPC specimens in ANSYS: (**a**) mesh of the SMP specimen in isometric view; (**b**) boundary conditions applied to the SMP specimen; (**c**) fine mesh of the SMPC specimen in isometric view; and (**d**) boundary conditions applied to the SMPC specimen. The arrow signifies the direction of the applied load.

A CAD model of a dog-bone specimen (ASTM D638 Type V) was created to represent the SMP, while a rectangular laminate specimen (ASTM D3039) was modeled for the SMPC. These geometries, commonly employed in universal testing machine (UTM) tensile tests, were generated in Fusion 360 and subsequently imported into ANSYS. To ensure high accuracy, a structured mesh technique was employed. Two boundary conditions were placed to emulate the tensile test to be conducted by the UTM. First, one end of the sample was fixed with zero displacement in all directions. Second, a displacement load was placed at the opposite end of the sample. Figure 4 illustrates the finite element model setup of the specimens in ANSYS.

The Static Structural Mode was used to simulate the deformation of the samples. Stress–strain and force–displacement curves were extracted to calculate the Young’s Modulus and determine the tensile strength of the material. The simulation results were then compared with the experimental values obtained from the mechanical characterization tests to validate the accuracy of the finite element model and ensure reliable predictions of the SMPC’s behavior.

### 2.5. Shape Memory Behavior Tests

Shape memory behavior tests were performed to assess the shape memory characteristics of the fabricated SMPCs, following the methodologies of Margoy et al. [15] and Bellisario et al. [16]. Among the three formulations, only SMPC-5 was selected for evaluation due to its enhanced storage and loss moduli. The remaining two formulations are analyzed in detail in a separate study by the authors.

**Figure 5:**
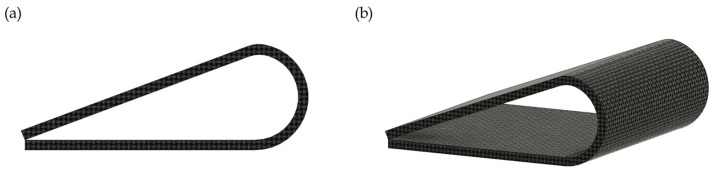
Front (**a**) and isometric (**b**) views of the SMPC-5 sample in its temporary configuration.

Two fundamental properties, shape fixity and shape recovery, were investigated over five thermomechanical cycles. The shape memory response was characterized by first heating the SMPC-5 sample above its glass transition temperature and into a hinge-like configuration with a 20° bending angle (see Figure 5). The specimen was then reheated to return to its original shape. This procedure was repeated five times, and the bending curvature was measured for each cycle. The same programming method was applied to quantify the shape recovery ratio.

The shape fixity ratio (Rf) was determined following Kumar et al. [17] and calculated using the equation:

(6) $$R_{f}\;(\%)=\frac{k_{nl}}{k_{p}}\;\times \;100$$

where *k_nl_* represents the resulting curvature exhibited by the SMPC after programming and subsequent release, and *k_p_* refers to the programmed or ideal bending curvature defined by the mold.

The shape recovery ratio *R_r_* was quantified using:

(7) $$R_{r}\;(t)\;=\;\frac{\theta_{(t)-}\theta_{start}}{\theta_{full}-\theta_{start}}\;\times \;100$$

where *θ_start_* corresponds to the angle measured immediately before recovery, *θ_full_* is the angle after full recovery, and *θ*(*t*) is the angle at time *t*. A ratio of 100% indicates full recovery of the sample’s original configuration.

## 3. Results and Discussion

### 3.1. Material Characterization

#### 3.1.1. Dynamic Mechanical Analysis (DMA)

DMA was conducted to evaluate how incorporating polyethylene glycol (PEG-600) influences the thermomechanical behavior of epoxy-based SMPCs reinforced with a single carbon–aramid fiber layer. The glass transition temperature (T_g_) of each sample was determined from the peak of the tan δ curve. As summarized in Table 3, the SMPC samples showed a decrease in T_g_ with increasing PEG-600 content. The neat composite (SMPC-P) exhibited a T_g_ of 89.79 °C, while the incorporation of 5 wt.% and 10 wt.% PEG-600 reduced the T_g_ to 70.28 °C and 59.34 °C, respectively. This downward shift in T_g_ corroborates the plasticizing effect of PEG, which disrupts the hydrogen bonding and reduces the crosslink density within the epoxy network [18]. Similar T_g_ reductions have also been observed in other shape memory polymer systems, such as cyanate esters [19] and polyurethanes [20].

Moreover, a comparison of T_g_ differences reveals that the drop from SMPC-P to SMPC-5 (ΔT_g_ = 19.51 °C) is more pronounced than the decrease between SMPC-5 and SMPC-10 (ΔT_g_ = 10.94 °C). This suggests that the effect of PEG on lowering T_g_ becomes less significant at higher concentrations and could be attributed to the saturation of the polymer matrix’s capacity to accommodate additional PEG molecules as the polymer chains have already achieved maximum mobility. The additional PEG molecules no longer contribute as effectively to chain separation and increased free volume. This highlights the non-linear relationship between plasticizer concentration and the resulting changes in T_g_. At lower PEG concentrations, on the other hand, the polymer chains have ample free volume to contribute to the PEG molecules, leading to a significant drop in T_g_ [21].

**Figure 6:**
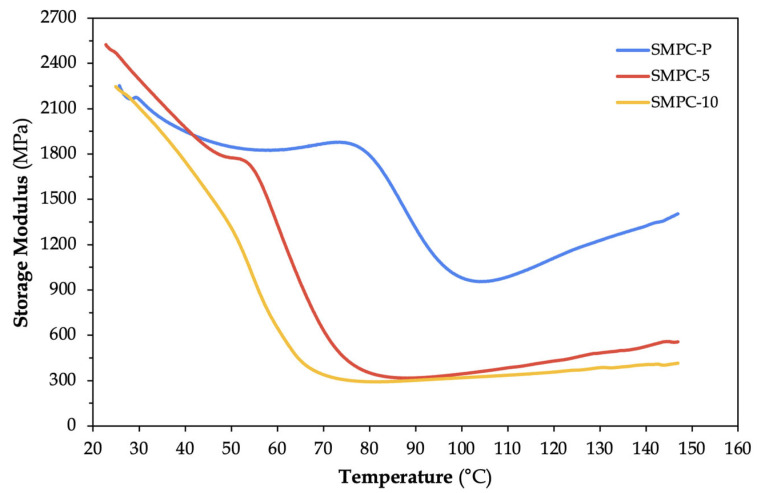
Storage modulus versus temperature graph of SMPC specimens with different PEG content.

The storage modulus (E′) is a crucial metric for quantifying the elastic response of viscoelastic materials [22], representing its stiffness in the elastic region. For shape memory polymer composites (SMPC), this value is typically measured at room temperature to reflect the material’s glassy state. As shown in Figure 6, the storage modulus of SMPC-5 (2444 MPa) is higher than that of the neat SMPC-P (2253 MPa). This initial increase indicates that the addition of 5 wt.% PEG-600 acts more like a cross-linker or filler. During curing, the short PEG-600 chains can interact with the epoxy network through hydrogen bonding or other secondary forces, effectively tightening the structure and increasing its cross-link density. Such “antiplasticization” effects have been reported in the literature, where the small molecules that strongly interact with the polymer matrix can both reduce T_g_ and increase modulus by densifying the network [23,24]. At this low concentration, the PEG-600 likely acts as an interfacial modifier. However, in SMPC-10, the storage modulus decreased to 2226 MPa and returned closer to the value of the unmodified composite. This implies that excessive plasticization may increase chain mobility and offset the initial reinforcing effect of PEG.

As the temperature increases, all three samples exhibit a sharp drop in storage modulus near their respective glass transition regions. This is a fundamental characteristic of polymers as they transition from a glassy to a rubbery state. Among the samples, SMPC-10 showed the earliest and most gradual decline in storage modulus. This behavior can be attributed to the higher PEG-600 content in SMPC-10 compared to SMPC-P and SMPC-5 and is consistent with the expected response of materials subjected to a greater degree of plasticization [25]. In such materials, reduced intermolecular interactions facilitate greater chain mobility. The observed trends highlight the importance of identifying the optimum concentration of PEG-600 to improve both flexibility and stiffness of the SMPC without sacrificing its structural integrity.

**Figure 7:**
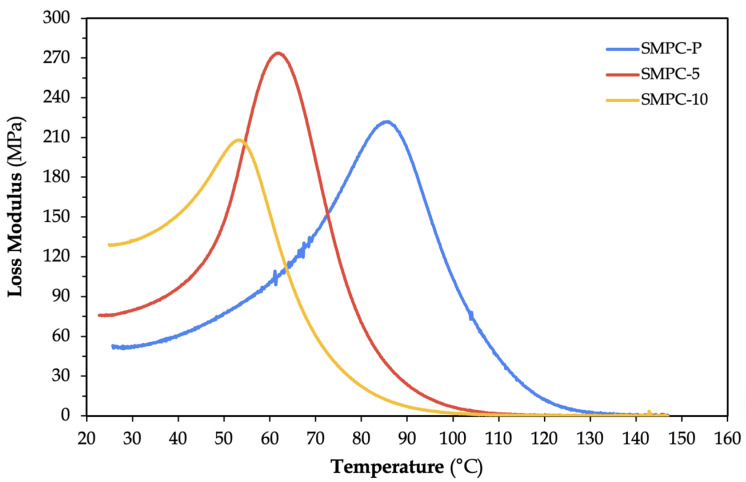
Loss modulus versus temperature graph of SMPC specimens with different PEG content.

The loss modulus (E″) quantifies the energy dissipated as heat due to internal friction from the movement of polymer chains within the material [26]. Figure 7 shows that the loss modulus increased from 222 MPa (SMPC-P) to 273.8 MPa (SMPC-5) after incorporating 5% of PEG-600.

The resulting increase in E″ suggests more efficient viscoelastic energy dissipation, likely due to a combination of increased chain mobility and sufficient matrix reinforcement. On the other hand, the lowest E″ value observed in SMPC-10 (208.1 MPa) may be attributed to reduced matrix–fiber interaction or potential phase separation. These observations suggest that while moderate PEG addition can enhance damping and thermal response, excessive PEG introduces a plasticizing effect that weakens the fiber–matrix interaction, thereby reducing mechanical strength and modulus [27].

**Figure 8:**
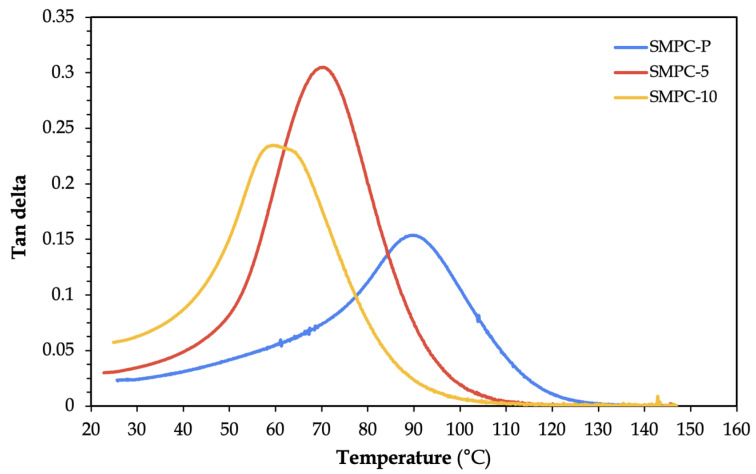
Tan delta versus temperature graph of SMPC specimens with different PEG content.

Figure 8 illustrates similar order of peaks between the loss modulus and tan delta curves. SMPC-5 obtained the highest tan delta value (tan δ = 0.3049), while the SMPC-P obtained the lowest (tan δ = 0.1539). The enhanced damping capacity in SMPC-5 is advantageous for actuation applications, indicating better energy dissipation and shape recovery potential.

It can also be observed that SMPC-P exhibited the broadest glass transition range, suggesting a more heterogeneous molecular environment typical of unmodified epoxy networks. In contrast, SMPC-5 showed the sharpest and most defined tan delta peak, indicating a more uniform polymer relaxation behavior due to partial PEG plasticization. Lastly, SMPC-10 showed a slightly broader transition, which may reflect partial phase separation or less uniform PEG dispersion. These results suggest that PEG incorporation was an effective strategy for tuning the thermomechanical properties of epoxy/carbon fiber SMPCs. The incorporation of 5 wt% of PEG in the SMPCs offered improved damping characteristics, broader thermal transitions, and lower actuation temperatures. These characteristics are favorable for applications requiring higher flexibility, energy absorption, and faster shape recovery. Additionally, the integration of carbon fibers played a crucial role in shaping thermomechanical behavior by constraining polymer chain mobility and facilitating efficient stress transfer between the matrix and the reinforcement. These findings further validated the potential of PEG as a molecular design component to optimize SMPC performance across diverse functional applications.

It is important to note, however, that the DMA instrument used in this study operated only between 20 °C and 160 °C, and as a result, measurements could not be taken in the sub-zero glassy region. The curves therefore started just before the glass transition and captured only the glass transition and rubbery regions. This limitation is acknowledged, and future work should employ equipment capable of measuring into the sub-zero range (−50 °C) to better characterize the glassy regime of the SMPCs.

#### 3.1.2. Fourier Transform Infrared Spectroscopy (FTIR)

The SMP and SMPC specimens were analyzed using FTIR to provide insight into their chemical structures by identifying characteristic absorption peaks associated with specific molecular vibrations. A summary of the observed peaks is presented in Table 4 below, while the corresponding overlaid spectra are shown in the accompanying figure.

**Figure 9:**
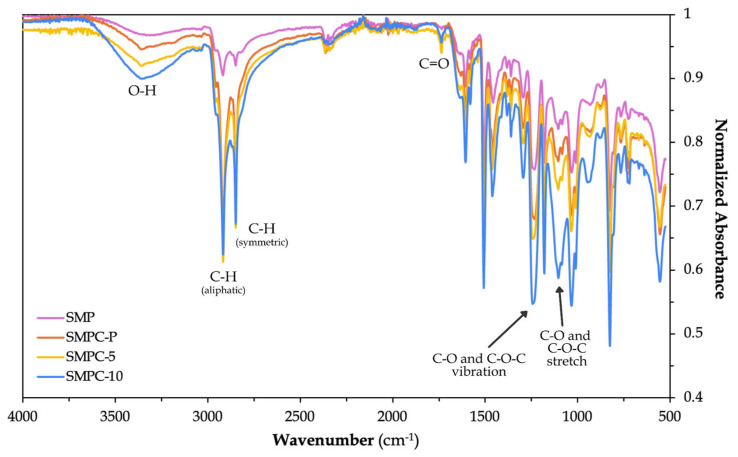
Normalized FTIR spectra of neat SMP and SMPC samples (SMPC-P, SMPC-5, and SMPC-10).

The observed peaks at above ~3300 cm^−1^ are exhibited by the polymer and composites which indicate the presence of hydroxyl (-OH) groups [28]. When carbon–aramid fiber was incorporated, a shift (i.e., from ~3312 cm^−1^ to ~3360 cm^−1^) in the hydroxyl peak was observed, which may have stemmed from Van Der Waals’ intermolecular forces between the fibers and the unbound hydroxyl groups of epoxy and/or hydrogen-bound epoxy–PEG molecules. The addition of fibers may also have led to the formation of an interfacial layer between the epoxy and/or epoxy–PEG and the fibers, where a different local environment is present in the interfacial region (i.e., -OH groups can now interact with the carbon–aramid surface) compared to when only pure polymer chains are present. Figure 9 presents the overlay of normalized FTIR spectra, which illustrates the spectral variations associated with fiber addition and the resulting interfacial interactions.

Moreover, the -O-H peak for SMPC-10, regarded as the composite with the highest PEG-600 content, showed a broader peak, which can be attributed to a stronger and more diverse hydrogen bonding network contributed by the PEG molecules. This allows hydrogen bonding between the hydroxyl groups of PEG and epoxy. As such, PEG serves as an additional source of hydrogen bonding in the network of the composites [29]. Hydrogen bonding increases due to the hydroxyl end groups of PEG, promoting chain termination or causing the development of fewer epoxy crosslinks, thereby lowering the network rigidity and glass transition of the composites, as supported by the DMA results.

The following column in the table demonstrates the sharpening or intensification of peaks linked to aliphatic C-H stretching vibrations (i.e., ~2920 cm^−1^ and ~2850 cm^−1^) after the integration of the carbon–aramid fibers [30]. This could result from polymer rearrangements during the curing or post-curing processes influenced by the fibers, leading to greater exposure of C-H groups near the surface. Such rearrangements modify the epoxy chain orientation or alignment near the fibers of the mat due to surface interactions between the epoxy and the carbon–aramid mat surface. Van der Waals forces (weak attractions that help guide molecular positioning) or π–π stacking of the benzene rings in the epoxy with the π-electron-rich surface of the carbon [31] may contribute to this effect, where the configuration could strengthen the alignment of CH_2_ dipoles along the direction of the infrared beam.

In addition to these changes, the fifth column highlights the emergence of carbonyl groups (C=O) with peaks at ~1740 cm^−1^, tentatively associated with the introduction of the carbon–aramid mat layer into the polymer matrix. Since these carbonyl groups were absent in the pure SMP, initial speculations emphasized the probable role of the carbon–aramid mat in promoting their formation. This may possibly suggest strong interfacial interactions between the epoxy and/or epoxy–PEG and the oxidized carbon surface, due to the formation of new ester or carbonyl linkages. Such links may have occurred when epoxy groups react with surface -COOH or -OH on the carbon fibers or as a result of catalytic effects provided by the carbon. This tentative interpretation can be confirmed in follow-up studies with the consideration of other possible attributions of this peak relating to oxidized polymer segments, initial fiber surface functional groups, or other matrix-related phenomena.

Peaks at ~950 cm^−1^ and ~830 cm^−1^ were linked to the -C-O- stretching and -C-O-C- stretching of the oxirane ring, respectively. These are characteristics of the epoxy resin, reflecting the chemical bonds that form its molecular structure [32]. In addition, peaks between 500 cm^−1^ and 1600 cm^−1^ appeared in all samples, associated with functional groups of the epoxy and/or the curing agent.

### 3.2. Mechanical Characterization

#### 3.2.1. Uniaxial Tensile Tests

##### Tensile Strength and Stiffness Analysis

**Figure 10:**
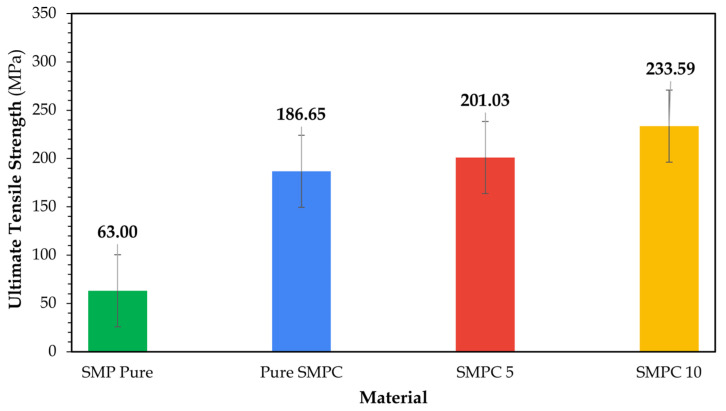
Ultimate tensile strength of neat SMP and SMPC samples (SMPC-P, SMPC-5, SMPC-10).

The ultimate tensile strength values were extracted from the raw stress–strain curves following standard ASTM procedures, taken as the maximum stress recorded before fracture. Figure 10 below depicts the variation in UTS across the four samples: neat SMP, SMPC-P, SMPC-5, and SMPC-10.

An increasing trend is evident, with neat SMP recording 63 MPa, SMPC-P reaching 187 MPa, SMPC-5 at 201 MPa, and SMPC-10 achieving 234 MPa. This represents an approximate 296% improvement from neat SMP to SMPC, underscoring the significant strengthening effect of fiber reinforcement.

The UTS of about 234 MPa for the SMPC under low fiber loading in the current study can be comparable to the aramid/epoxy composites with seven layers of fiber and resin matrix content of about 20% from the study by Xiao et al. [33] for their phthalic anhydride modified aramid fiber plain fabric/epoxy composites (231.9 MPa). The UTS for carbon or aramid prepreg composites with a quasi-isotropic layup and a 50/50 component ratio may reach beyond 600 MPa for the aramid and 2000 MPa for the carbon [34]. The recorded UTS in the present study may suggest the contribution of other factors beyond fiber content. Effective stress transfer, good interfacial adhesion, and PEG-induced matrix toughening may have enhanced the strength performance.

Pure epoxy resin systems often display mechanically inferior properties in their unreinforced states. Kumar et al. [35] imply that this substance is prone to brittle fracture under tensile loading and poor stress distribution as no other reinforcements carry the tensile load. Due to its sensitive nature in the fabrication process, most matrices are prone to the formation of entrapped air, which weakens the material by initiating crack propagation and further hindering proper load distribution. In contrast, carbon fibers, due to their highly ordered graphite lattice, in which atoms are parallel to the fiber axis, contribute exceptionally high specific modulus and strength, which they can sustain at elevated temperatures, while aramid fibers provide low weight and high impact resistance [36]. A study by Eremin et al. [37] showed that the ultimate tensile stress of aramid fibers can reach up to 620 MPa, while carbon fibers can reach values of 1098 MPa in an orthotropic [0/90] layup, similar to the configuration used in this study, reinforcing the effect seen here. While the strength contributions from aramid fibers are relatively lower due to their increased flexible molecular structures, reinforcement with carbon fibers significantly enhances the overall mechanical strength.

For all composite samples, the addition of PEG further increased strength. SMPC-5 and SMPC-10 exhibited higher UTS values than SMPC-P, with 10 wt.% PEG providing the maximum improvement. These trends agree qualitatively with Singh et al. [25] wherein the increase in weights of PEG-400, incorporated into epoxy systems, improved bonding between both networks which improved fracture toughness significantly. In this study, the addition of PEG-600 likely introduced the same effect, enhancing flexibility and plasticity of the epoxy matrix and possibly improving interfacial adhesion with carbon and aramid fibers, with a 10 wt.%. PEG was considered as the threshold limit in this study, which balanced strength and flexibility, without compromising structural integrity.

##### Young’s Modulus and Structural Stiffness

**Figure 11:**
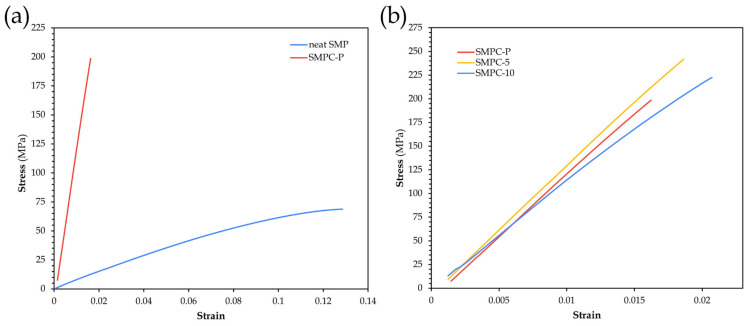
Stress–strain curves of neat SMP and SMPC-P samples (**a**) and SMPC (SMPC-P, SMPC-5, SMPC-10) samples (**b**).

Under axial tensile loading, the force versus extension is converted into a stress–strain curve to quantify how much a material deforms prior to plasticity. The slope in the linear region serves as the basis for calculating stiffness. Figure 11 presents the mechanical response of SMP Pure and SMPCs.

SMP Pure exhibited a slow, sustained strain path, from gradual elongation to a curved path before fracture, while neat SMPC exhibited a sharp rise before ending abruptly, manifesting a higher slope, therefore, a higher elastic modulus. This improvement in Young’s modulus reflects the dominance of fiber reinforcement, with mass fractions of 0.85 implying that properties are ruled by woven reinforcements, consistent with the literature reporting carbon fiber-reinforced composites reaching 54.1 GPa and carbon–aramid fiber composites 48.5 GPa [36].

Given the [0/90] alignment, the material can bear loads in both longitudinal and transverse directions, which makes it beneficial for tensile loading. A study by Sakhawat et al. [38] revealed that fiber orientation and volume fraction significantly influence the elastic modulus of composites, with maximum stiffness at a fiber inclination angle of 0°, when fibers are positioned along the longitudinal axis, while it remains constant beyond 45° as off-axis fibers contribute less. The combination of carbon and aramid fibers in a [0/90] twill weave offers a balanced effect that optimizes their favorable characteristics to create a composite that maximizes elastic modulus while maintaining good ductility and structural resilience.

Among SMPCs, PEG influenced modulus differently than strength. All samples maintained near-perfect linear behavior, indicating elastic response up until failure. SMPC Pure was found to have the lowest elastic modulus among the three, SMPC-5 exhibited the steepest slope, and thus highest modulus, while SMPC-10, despite reaching the highest UTS, showed slightly reduced stiffness. Similar trends aligned with the findings of Singh et al. [25], who reported increases of up to 38.5% in tensile strength and 37.8% in tensile modulus with the addition of PEG-10. These improvements were attributed to the disruption of normal curing conditions, which enhanced molecular dynamics among epoxy crosslinked polymers, resulting in a more structured internal stress distribution under quasi-static tensile loading.

SMPC-5 likely achieved the highest tensile modulus due to achieving an optimal balance between improved adhesion of the epoxy network to the fibers and avoiding the excessive plasticizing effects observed in SMPC-10. Moreover, in this study, although PEG-10 is considered the threshold for maintaining favorable mechanical conditions, it may not be optimal here due to excessive soft segment formation, which likely reduced the overall stiffness despite improved strength [25].

##### Failure Modes

Visual inspection of the SMPC laminates after hand layup and post-curing revealed no evidence of phase separation or insufficient fiber wetting. To evaluate the tensile behavior of the composites, failure modes were analyzed based on elongation and strain at break (Table 5) as well as fracture morphology (Figure 11). SMP Pure exhibited the highest elongation (11.225%) and strain at break (0.112), with uniform breaks across samples.

**Figure 12:**
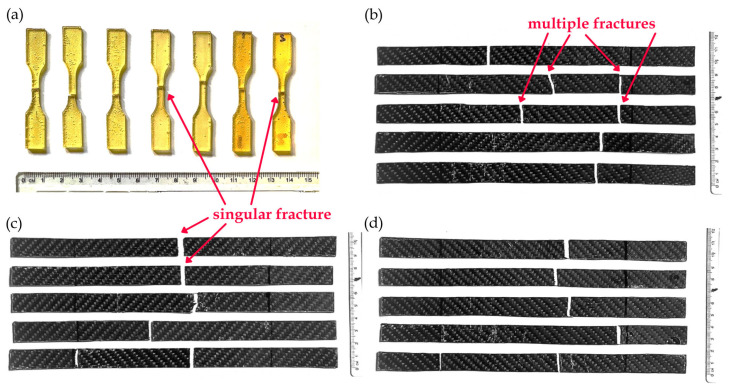
Representative fracture surfaces of (**a**) neat SMP, (**b**) SMPC-P, (**c**) SMPC-5, and (**d**) SMPC-10 after UTM tensile testing.

From Table 5, a clear decrease in elongation and strain at break is observed when transitioning from SMP Pure to the fiber-reinforced composites, confirming the more brittle behavior induced by fiber reinforcements. SMP Pure exhibited the highest elongation and strain at break, while the composites were remarkably lower. Notably, compared with SMPC-P and SMPC-5, SMPC-10 exhibited a small but noticeable recovery in elongation and strain at break, reflecting the influence of PEG in enhancing ductility. These fractures are visually represented in Figure 12.

The neat SMP is depicted with singular, near-uniform breaks across samples. In contrast, SMPC-P and SMPC-5 exhibited multiple, cleaner, straighter breaks with minimal deformation, while SMPC-10 displayed slightly more irregular fracture lines. These observations align with Singh et al. [25], who reported that the addition of PEG decreased viscosity and improved failure strain by up to 54% compared to neat epoxy. This may be attributed to PEG acting as a plasticizer, increasing the formation of soft segments within the polymeric network and allowing for more extensible deformation, possibly before fracture, overcoming the rigidity of fiber reinforcements. The addition of woven material significantly increases the overall strength and stiffness, creating a trade-off between a stiffer, more rigid body and the polymer’s ability to deform. This rigidness is often the reason stress is more concentrated, allowing fracture to happen more quickly than in their unreinforced SMP states.

Results show that, in general, fiber reinforcement and PEG incorporation substantially enhanced the mechanical properties of SMPs. SMPC-5 demonstrated the highest tensile modulus and stiffness, while SMPC-10 exhibited the greatest UTS. Although SMP Pure showed the highest elongation, the robust performance of SMPCs makes them suitable for reliable structural applications.

#### 3.2.2. ANSYS Simulations

ANSYS simulations were conducted to assess how the primary constituents of the shape memory polymer composite influence its mechanical performance, with particular focus on the Young’s modulus. Prior to the simulations, the material properties of epoxy resin [14], carbon fiber [39], and aramid fiber [40] were taken from literature, as summarized in Table 6. The tensile modulus used for the SMP simulation, however, was based on experimental results. Kevlar-49 was also considered as the representative aramid fiber for the property inputs. It should be noted that the hybrid composite could not be directly simulated due to the limited availability of orthotropic properties data representing carbon–aramid composites; hence, the reinforcing fibers were modeled separately to demonstrate individual contributions.

**Figure 13:**
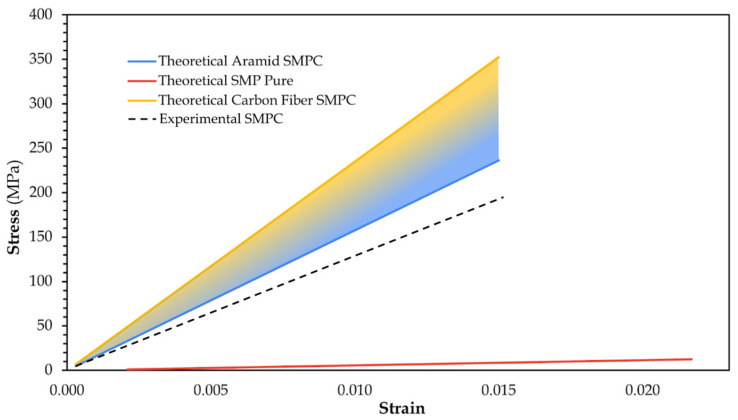
Theoretical stress–strain curves of neat SMP, carbon fiber SMPC, and aramid SMPC, and experimental stress–strain curve of the actual SMPC.

The simulated values are close to the experimental findings from UTM testing, where the hybrid SMPC achieved an average Young’s modulus of ~13 GPa across all datasets. To illustrate the trends, stress–strain curves were generated from simulation of carbon fiber SMPC, aramid fiber SMPC, and SMP Pure, with experimental SMPC results superimposed, as shown in Figure 13.

From the plot, carbon fiber SMPC clearly exhibited the highest stiffness corresponding to a modulus of approximately 23 GPa. Aramid fiber SMPC followed with a modulus of 16 GPa, while SMP Pure displayed a much lower modulus of only about 580 MPa. This large discrepancy reinforces the highly ductile nature of SMPs compared to reinforcing fibers. The observed modulus gap can be attributed to the fundamental roles of fibers in composite structures, where they primarily act as the load-bearing elements, while the polymer matrix provides binding and facilitates stress transfer and overall deformation [41].

These simulation results were validated against experimental UTM testing results of SMP Pure and SMPC Pure. SMP Pure, tested independently, recorded a Young’s modulus of 760 MPa and a UTS of 63 MPa. On the other hand, SMPC Pure, containing SMP, aramid, and carbon fiber, achieved a significantly higher modulus of 13 GPa and a UTS of 187 MPa. These results confirm that fiber reinforcement considerably enhanced both stiffness and strength relative to the pure polymer matrix. However, it was observed that the experimental SMPC Pure has lower modulus than the expected range of 16–23 GPa for the carbon–aramid hybrid SMPC. This suggests two possibilities: (1) the actual fiber volume fraction is lower than expected, as the composite’s modulus is sensitive to fiber volume fraction and (2) the hybrid fiber is dominated by aramid fiber, which is of lower modulus than carbon fiber.

Overall, the comparison highlighted the effectiveness of hybridization in combining stiff fibers with a flexible matrix. While the SMP provides ductility and enables shape memory functionality, the addition of carbon fiber for strength and aramid fiber for toughness ensures significant mechanical reinforcement, making the composite well-suited for applications that demand both structural reliability and recoverability.

### 3.3. Shape Fixity and Recovery

**Figure 14:**
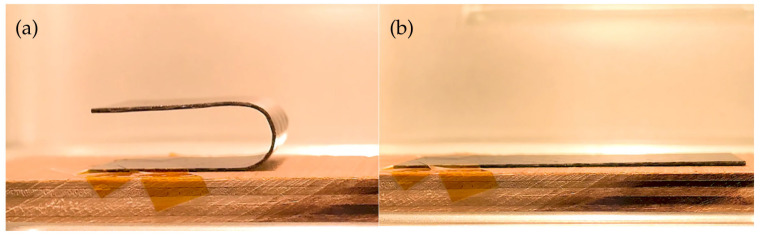
Shape memory behavior of SMPC-5: (**a**) programmed (deformed) configuration and (**b**) recovered configuration.

To illustrate the shape memory response, representative images of SMPC-5 in its programmed (deformed) and recovered states are presented in Figure 14. These images serve as a visual reference for the subsequent analysis of shape fixity and recovery behavior. SMPC-5 was selected as an initial validation sample to demonstrate the response. The effect of varying PEG-600 content on the shape fixity and recovery behavior of the composites has been discussed in a separate research paper by the same author.

The shape fixity behavior of SMPC-5 is summarized in Table 7. In the first two cycles, the shape fixity remained consistent at 98.45% before experiencing a gradual decrease in the succeeding cycles. The sample showed only a slight decline from cycles 2 to 4 but then dropped more noticeably to 89.44% in the fifth cycle. This trend indicates that in the case of SMPC-5, repeated thermo-mechanical cycling may reduce the ability of the composite to retain its temporary shape. However, the decline (ΔR_f, 1→5_ = 9.01%) observed here is considerably larger than expected. For comparison, Li et al. [11] reported only a 2.6% reduction in shape fixity (from 98.3% to 95.7%) for carbon fiber–reinforced SMPCs after 30 cycles. This discrepancy suggests that the current system may still be optimized through adjustments in the epoxy matrix formulation or fiber–matrix interfacial bonding. In addition, exploring different bending angle configurations may further improve fixity performance since programming strain levels directly affect shape retention. Despite this relatively larger decline, SMPC-5 still maintained a relatively high level of shape retention, with an average fixity ratio of 95.76 ± 3.85%. This indicates that the composite retains functional reliability under the present testing conditions, while leaving room for further optimization.

**Figure 15:**
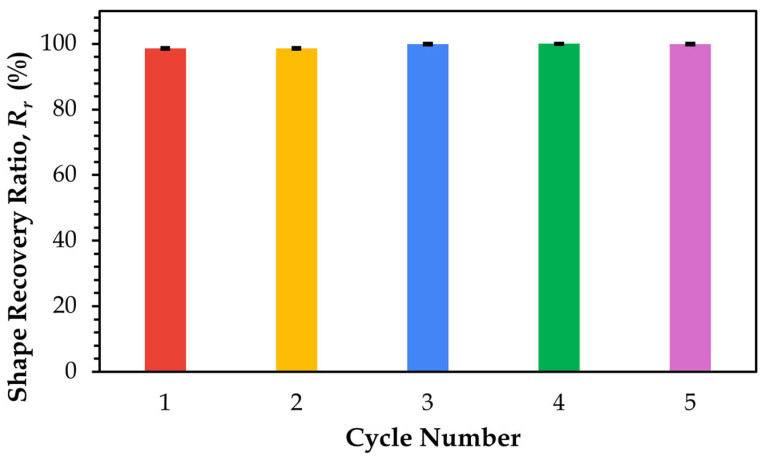
Shape recovery ratio (%) of SMPC-5 in 20° bending angle configuration across 5 thermomechanical cycles.

The shape recovery ratio of SMPC-5, plotted as a function of time across five thermomechanical cycles, is shown in Figure 15.

In the graph above, as cycling progressed, the recovery values stabilized near complete recovery, increasing from 98.63% in Cycle 1 and 98.60% in Cycle 2 to 99.90% in Cycle 3, reaching 100% in Cycle 4, and slightly decreasing to 99.89% in Cycle 5. These values are consistently close, with only minor variations, and demonstrate overall stabilization. Notably, Cycle 5 exhibited the fastest recovery among all samples, suggesting that repeated thermomechanical cycling progressively enhances recovery speed as the material adapts through internal structural modifications. A similar trend was observed by Li et al. [11], who studied recovery in unidirectional carbon fiber-reinforced SMPCs. In their work, early cycles showed an initial recovery phase associated with greater decrease in stiffness loss and loss factors, indicating material adaptation. After approximately three to ten cycles, these variables stabilized, resulting in a more consistent and efficient recovery. These observations support the notion that recovery performance improves with repeated cycling due to structural stabilization and adaptability. The addition of PEG may also affect recovery performance. This plasticizer increases chain mobility, facilitating an accelerated glassy-to-rubbery transition, thereby enabling more efficient shape recovery during successive cycles [10].

Moreover, for high-temperature applications of SMPCs, polyethylene glycol (PEG) can compromise performance due to several key factors. At elevated temperatures, particularly near or above its melting point, PEG may leach out of the composite, especially when present in high concentrations or with low molecular weight, thereby reducing structural integrity and functionality. High temperatures can also induce phase separation between PEG and other polymer components as a result of limited miscibility, leading to inhomogeneous properties and decreased reliability in shape memory cycles. While PEG enhances flexibility at moderate temperatures, its excessive presence can lower the overall thermal stability of SMPCs. Prolonged exposure to high temperatures further accelerates thermal degradation, ultimately resulting in the loss of shape memory properties and structural failure.

## 4. Conclusions

This study characterized fiber-reinforced shape memory polymer composites (SMPCs) with tunable glass transition temperatures (T_g_) achieved through the incorporation of PEG-600. Dynamic Mechanical Analysis (DMA) confirmed that PEG-600 effectively acts as both a plasticizer and molecular modifier, progressively reducing the T_g_ to potentially enable controlled thermal actuation essential for sequential and time-dependent deployment systems. At 5 wt.%, PEG enhanced storage and loss moduli, whereas higher loading (10 wt.%) introduced excessive plasticization that compromised stiffness. FTIR analysis further validated these effects by revealing shifts in hydroxyl, aliphatic C–H, and carbonyl peaks, indicating hydrogen bonding and interfacial interactions within the epoxy–PEG–fiber system.

Uniaxial tensile testing showed that fiber reinforcement significantly improved the mechanical performance of the SMPCs. SMPC-10 exhibited the highest ultimate tensile strength at 233.59 MPa, while SMPC-5 showed the highest Young’s modulus of 14.081 GPa. These improvements confirmed that the hybrid reinforcement of carbon and aramid fibers enhanced the structural integrity of the composite, making it suitable for load-bearing applications. Looking forward, optimizing PEG content and fiber configurations remains critical to fully exploit the trade-offs between flexibility and strength. Future investigations should also address multilayer arrangements and performance under sub-zero conditions to further validate glassy-state behavior. Collectively, these directions will support the development of smart composites with reliable, programmable actuation and structural performance across engineering applications.

## Figures and Tables

**Table 1:** The material specifications for components A and B of the epoxy resin.

Properties	Component A (Resin)	Component B (Hardener)
Specific gravity (at 30 °C)	1.08 gm/ml	0.915 gm/ml
Density (at 25 °C)	1.16 g/cm^3^	-
Boiling point	>100 °C	
Shore D Hardness	83	
Tensile strength of cured epoxy resin	6500–8000 psi	
Flexural strength of cured epoxy resin	13,500–15,500 psi	

Note: Data adopted from Polymer Products (Phils.) Inc.

**Table 2:** The material specifications for carbon fiber reinforcement.

Properties	Aramid (Primary Fiber)	Carbon (Secondary Fiber)
Weave pattern	2 × 2 Twill	
Fiber orientation	0°/90°	
Areal density	238 g/m^2^	
Thickness	0.43 mm	
Tensile strength	424 ksi	512 ksi
Tensile modulus	10.2 msi	33.4 msi
Strain to failure	3.6%	1.5%
Density	1.44 kg/L	1.80 kg/L

Note: Data adopted from Polymer Products.

**Table 3:** Summary of the viscoelastic properties of the three SMPC formulations.

Sample	Storage Modulus, E′ (MPa)	Peak Loss Modulus, E″ (MPa)	Glass Transition Temperature, T_g_ (°C)	∆T_g_
SMPC-P	2253	222.0	89.79	-
SMPC-5	2444	273.8	70.28	19.51
SMPC-10	2226	208.1	59.34	30.45

**Table 4:** FTIR spectral peak comparison of SMP and SMPC samples (SMPC-P, SMPC-5, SMPC-10) with functional group assignments.

Sample	Corresponding Functional Groups to Identified Peaks (cm^−1^)				
	Hydroxyl (-O-H)	Aliphatic C-H (Asymmetric)	Aliphatic C-H (Symmetric)	Carbonyl (C=O)	-C-O- and -C-O-C- Stretching
SMP	3312.52	2918.22	2849.04	Absent	Similar peaks (1600–500 cm^−1^)
SMPC-P	3359.77	2916.52	2848.38	1736.61	
SMPC-5	3355.21	2916.90	2848.61	1735.77	
SMPC-10	3360.43	2917.04	2848.76	1736.29	

**Table 5:** Elongation and strain at break of neat SMP and SMPC samples (SMPC-P, SMPC-5, SMPC-10).

Sample	Elongation (%)	Strain at Break
SMP Pure	11.225 ± 1.386	0.112 ± 0.014
SMPC-P	1.618 ± 0.263	0.016 ± 0.003
SMPC-5	1.560 ± 0.183	0.016 ± 0.002
SMPC-10	2.068 ± 0.232	0.021 ± 0.002

**Table 6:** Engineering Data (Orthotropic) of Carbon Fiber and Aramid Fiber Composites.

Material Specification	Carbon Fiber	Aramid Fiber
Young’s Modulus X Direction	23714.533 MPa	15,856.043 MPa
Young’s Modulus Y Direction	23714.533 MPa	15,856.043 MPa
Young’s Modulus Z Direction	843.9524828 MPa	842.4665459 MPa
Poisson’s Ratio XY	0.3406	0.2654
Poisson’s Ratio YZ	0.335	0.215
Poisson’s Ratio XZ	0.335	0.215
Shear Modulus XY	1172.026168 MPa	7584.973166 MPa
Shear Modulus YZ	1169.529598 MPa	6746.189024 MPa
Shear Modulus XZ	1169.529598 MPa	6746.189024 MPa

**Table 7:** Radius and corresponding bending curvature of SMPC-5 across five cycles.

Cycle No.	Radius of Curvature, *r_nl_* (cm)	Bending Curvature, *k_nl_* (cm^−1^)	Shape Fixity Ratio ^1^, *R_f_* (%)
1	0.645	1.5504	98.45
2	0.645	1.5504	98.45
3	0.650	1.5385	97.69
4	0.670	1.4925	94.78
5	0.710	1.4085	89.44
		Average *R_f_*	95.76 ± 3.85

^1^ Shape fixity ratio (%) = (*k_nl_*/*k_p_*) × 100, with *k_p_* = 1.5748 cm^−1^ obtained from the ideal programmed curvature.

## Data Availability

The original contributions presented in this study are included in the article. Further inquiries can be directed to the corresponding authors.

## Abbreviations

The following abbreviations are used in this manuscript:

ATR	Attenuated total reflectance
DGEBA	Bisphenol A diglycidyl ether
DMA	Dynamic mechanical analysis
FEA	Finite element analysis
FTIR	Fourier Transform Infrared Spectroscopy
T_g_	Glass transition temperature
LVR	Linear viscoelastic region
PEG	Polyethylene glycol
SMP	Shape memory polymer
SMPC	Shape memory polymer composites
UTS	Ultimate tensile strength
UTM	Universal testing machine
